# Zn/Cd ratios and cadmium isotope evidence for the classification of lead-zinc deposits

**DOI:** 10.1038/srep25273

**Published:** 2016-04-28

**Authors:** Hanjie Wen, Chuanwei Zhu, Yuxu Zhang, Christophe Cloquet, Haifeng Fan, Shaohong Fu

**Affiliations:** 1 State Key Laboratory of Ore Deposit Geochemistry (SKLODG), Institute of Geochemistry, Chinese Academy of Sciences, Guiyang, 550002, China; 2 Centre de Recherches Petrographique et Geochimiques, CNRS/UMR 7358, 15, Rue Notre-Dame-Pauvres, B. P. 20, 54501, Vandoeuvre-les-Nancy Cedex, France

## Abstract

Lead-zinc deposits are often difficult to classify because clear criteria are lacking. In recent years, new tools, such as Cd and Zn isotopes, have been used to better understand the ore-formation processes and to classify Pb-Zn deposits. Herein, we investigate Cd concentrations, Cd isotope systematics and Zn/Cd ratios in sphalerite from nine Pb-Zn deposits divided into high-temperature systems (e.g., porphyry), low-temperature systems (e.g., Mississippi Valley type [MVT]) and exhalative systems (e.g., sedimentary exhalative [SEDEX]). Our results showed little evidence of fractionation in the high-temperature systems. In the low-temperature systems, Cd concentrations were the highest, but were also highly variable, a result consistent with the higher fractionation of Cd at low temperatures. The δ^114/110^Cd values in low-temperature systems were enriched in heavier isotopes (mean of 0.32 ± 0.31‰). Exhalative systems had the lowest Cd concentrations, with a mean δ^114/110^Cd value of 0.12 ± 0.50‰. We thus conclude that different ore-formation systems result in different characteristic Cd concentrations and fraction levels and that low-temperature processes lead to the most significant fractionation of Cd. Therefore, Cd distribution and isotopic studies can support better understanding of the geochemistry of ore-formation processes and the classification of Pb-Zn deposits.

Lead-zinc (Pb-Zn) deposits are typically classified by their ore-formation processes and geological settings. Such deposits can be formed by the discharge of deep sedimentary brine onto the sea floor (sedimentary exhalative; SEDEX) or by replacement of limestone (Mississippi Valley type; MVT)^1^,^2^, whereas some are associated with submarine volcanoes (volcanogenic massive sulfide; VMS) or are found in the aureoles of sub-volcanic intrusions of granite (e.g., skarn and porphyry deposits)^3^. Tectonically, SEDEX deposits mainly form in passive margins or Proterozoic rift/sag basins, whereas MVT deposits form in carbonate platform sequences at passive margins^1^,^4^,^5^,^6^. Although the classification appears to be clearly defined, it remains difficult to implement in practice. For example, most deposits classified as SEDEX lack unequivocal evidence of an exhalite in the ore or an alteration component^7^. Although the presence of laminated sulfides parallel to bedding is usually accepted as evidence of an exhalative ore, some MVT deposits also contain extensive (kilometer-scale) laminated and stratiform ores formed by highly selective carbonate replacement millions of years after the deposition of the host rocks^1^. Furthermore, some vein-type Pb-Zn deposits associated with magmatism are difficult to recognize because the intrusion is often concealed. Indeed, many ore deposits may be formed by one or more of the basic processes mentioned above, thus resulting in ambiguous classifications and substantial controversy and conjecture. Therefore, much effort has been invested in the discrimination and classification of Pb-Zn deposits on the basis of geochemical proxies (e.g., fluid-inclusion studies and S-Pb-C-O-H isotopes). In recent years, new isotope systematics, such as Cd and Zn isotopes, have been used to better understand ore-formation processes^8^,^9^,^10^.

Cd has eight stable isotopes and is typically hosted in sphalerite in Pb-Zn deposits at concentrations of several hundred to several thousand ppm^11^,^12^. Many studies have shown that Cd isotope fractionation occurs when evaporation and condensation processes occur, resulting in δ^114/110^Cd values between −8‰ and +16‰ in meteorite samples^13^,^14^,^15^,^16^,^17^. Lacan *et al.*^18^ have studied Cd cycling in the ocean and have shown that phytoplankton preferentially take up light Cd isotopes from seawater. The biological fractionation of Cd isotopes (giving a total δ^114/110^Cd range of 4.4‰) in surface seawater has been found to be primarily governed by Rayleigh fractionation during the near-quantitative uptake of dissolved Cd by phytoplankton^18^,^19^,^20^,^21^,^22^. Significant variations in Cd isotopic ratios have been found in natural terrestrial samples with a total fractionation of approximately 1.5‰ in δ^114/110^Cd values for samples from mid-ocean ridge basalt (MORB) and ocean island basalt (OIB) rocks, loess, sediments, and sulfides^10^,^23^,^24^. Thus, different geological processes result in distinct Cd isotope fractionation, which might possibly be used to effectively track formation processes in ore deposit geology. In this paper, we report the systematic measurements of Cd isotopes in different types of Pb-Zn deposits and provide direct evidence for the effective use of isotopic studies in ore-formation processes and the classification of Pb-Zn deposits.

## Zn/Cd ratios and Cd isotopic compositions from different types of Pb-Zn deposits

**Figure 1:**
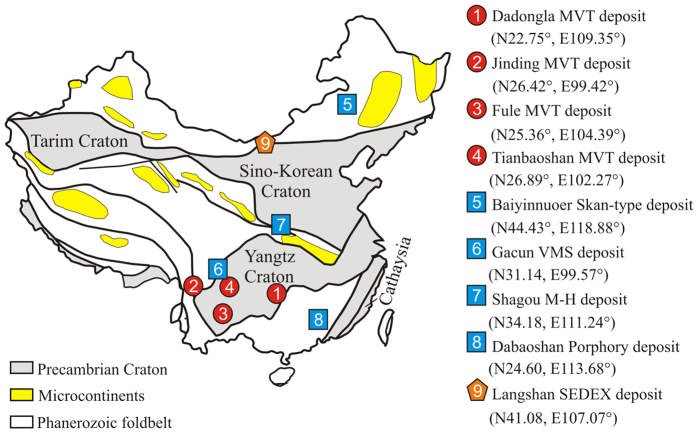
Locations of the Pb-Zn deposits chosen in this study, modified from ref. 25.

Nine Pb-Zn deposits in China were chosen, and their Cd isotopic compositions and relevant elemental ratios were investigated (Fig. 1)^25^. The deposits were divided into three categories: high-temperature systems, low-temperature systems and exhalative systems. Herein, the high-temperature systems referred to magma/volcanic-related deposits and included a porphyry deposit (Dabaoshan), magmatic hydrothermal deposit (Shagou), skarn deposit (Bayinnuoer) and VMS deposit (Gacun), all of which are closely associated with magmatism and formed at temperatures of approximately 200–250 °C^26^,^27^,^28^. The low-temperature systems referred to MVT deposits, including the Fule, Tianbaoshan, Jinding and Dadongla Pb-Zn deposits, which typically formed at temperatures <200 °C^29^. Finally, the exhalative systems were represented by the Langshan SEDEX deposit. The ore deposit geologies of these deposits are provided in the Supplementary materials.

**Figure 2:**
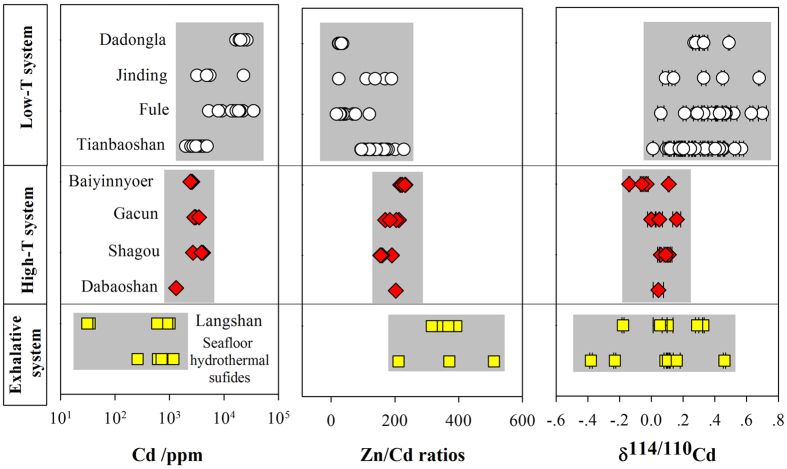
Distribution of Cd concentrations, Zn/Cd ratios and Cd isotopes in Pb-Zn deposits; reference data for seafloor hydrothermal sulfides from Schmitt *et al.*^24^.

The analytical data and elemental analyses are presented in Supplementary Table 3, and the elemental enrichment ratios (Zn/Cd) and δ^114/110^Cd values are illustrated in Fig. 2.

### High-temperature systems

Despite the existence of different ore deposit types (porphyry, magmatic hydrothermal, skarn, and VMS), the Cd concentrations, Zn/Cd ratios and Cd isotopic compositions were consistent. Cadmium concentrations from four deposits yielded a mean value of 2932 ppm and ranged from 2410 ppm to 4126 ppm, and the Zn/Cd ratios varied from 155 to 223 with a mean value of 195. No significant isotopic fractionation was found in these deposits, with values ranging from −0.14 to 0.16‰ and a mean δ^114/110^Cd of 0.04 ± 0.16‰ (2σ).

### Low-temperature systems

Samples from the four low-temperature MVT deposits showed distinct distributions compared with those from the high-temperature systems. Cadmium concentrations were highest but varied in the 2415–34981 ppm range and had a mean value of 9399 ppm. Zn/Cd ratios ranged from 17 to 201 and had a mean value of 101. The δ^114/110^Cd values were highly variable and enriched in heavier isotopes, exhibiting a mean value of 0.32 ± 0.31‰ (2σ, from 0.09 to 0.70‰).

### Exhalative systems

Sphalerite samples of the Langshan SEDEX deposit had the lowest Cd concentrations of the three categories and ranged from 595 to 996 ppm range (mean of 832 ppm) and exhibited tightly grouped Zn/Cd ratios in the 316–368 range (mean of 353). The deposit yielded an average δ^114/110^Cd value of 0.12 ± 0.50‰ (2σ) and ranged from −0.18 to 0.33‰. Measurements of seafloor hydrothermal sulfides, which are comparable to SEDEX deposits, revealed similar distributions with low Cd contents (259–1174 ppm, mean of 690 ppm) and high Zn/Cd ratios (211–510, mean of 366). The δ^114/110^Cd value is also typically highly variable, ranging from −0.38‰ to 0.46‰ (mean of 0.01 ± 0.27‰, 2σ)^20^.

### Thermodynamic simulation of the Cd content and Zn/Cd ratios in sphalerite

The differences in the Cd concentrations and Zn/Cd ratios in sphalerite from the three studied systems were consistent with those in previous investigations^11^. Statistical analysis of 480 Pb-Zn deposits suggested that exhalative deposits had low Cd concentrations (mean of 2560 ppm for SEDEX deposits), that MVT deposits (mean of 4850 ppm) and veins in carbonate rock (mean of 7260 ppm) had high concentrations, and that Skarn deposits (mean of 3540 ppm) had intermediate concentrations. Thermodynamics is a useful tool for explaining the variations in the Cd content of sphalerite from various systems with different mineralization processes.

Cadmium is mainly involved in direct Zn^2+^ ↔ Cd^2+^ substitutions in sphalerite; therefore, the distributions of Cd and Zn between the liquid and solid phases can be approximated by the following equilibrium involving the end-member compositions of CdS and ZnS^30^,^31^,^32^:


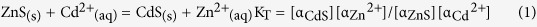


**Figure 3:**
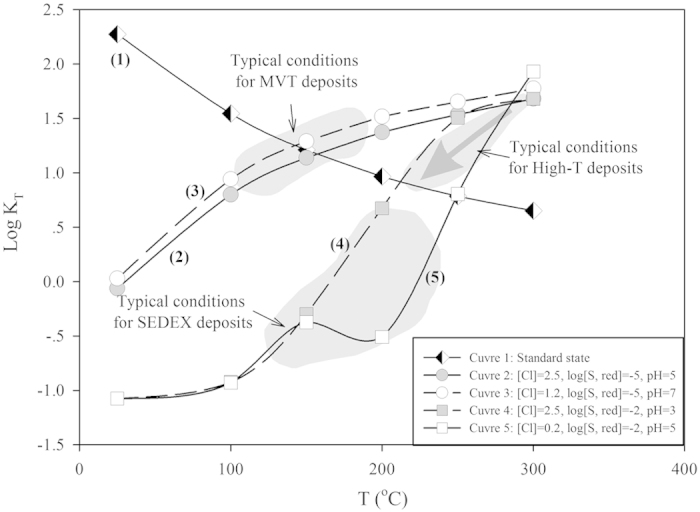
Zn-Cd partitioning between liquid and coexisting sphalerite, based on logK_T_ versus temperature under different hydrothermal conditions with Cl^−^ activity, total reduced sulfur, and pH. Calculated thermodynamic data were obtained from Schwartz (2010) and analyzed with SUPCRT92 software.

A rapidly decreasing trend was observed for the calculated thermodynamic distribution coefficient, K_T_, of this reaction (1) in the theoretical case of a complete absence of complexing ions as the temperature increases from 25 °C to 300 °C, as illustrated in Fig. 3 (Curve 1); thus, low temperatures favor Cd substitution in sphalerite. However, in hydrothermal fluids, most Zn and Cd exist as aqueous complexes, with Zn^2+^ and Cd^2+^ being subordinate^33^,^34^. The most important ligands are Cl^−^, HS^−^ and OH^−^ in hydrothermal fluids^33^,^35^,^36^. For example, aqueous Cd speciation is dominated by chloride species, CdCl_m_(H_2_O)_n_^2−m^, over a wide range of temperatures (20 ≤ T ≤ 450 °C), acidities (1 ≤ pH ≤ 8) and chloride concentrations (0.04 ≤ m_Cl_ ≤ 18 mol/kg H_2_O). Therefore, in addition to the temperature, the presence of Cd complexes, salinity (Cl^−^ concentration), concentration of reduced sulfur (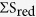) and pH value of hydrothermal fluids are also major factors that control the Cd distribution in sphalerite.

As shown in Curves 2–5 in Fig. 3, in contrast with Curve 1, this trend was reversed if the effect of complexing agents was considered. Curves 2 through 5 have several important implications. For hydrothermal fluids with similar reduced sulfur (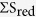) concentrations and pH values, there was no obvious difference in the distribution coefficient, K_T_, at the same temperature (see Curves 2 and 3 or 4 and 5), if only the salinity (Cl^−^ concentration) was considered. However, the reduced sulfur (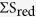) concentrations of ore-forming fluids affected the Cd distribution in sphalerite, whereas the salinity and pH did not. High 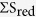 activities shifted K_T_ to lower values, favoring the formation of Cd-poor sphalerite (Curves 4 and 5 in Fig. 3), whereas low 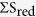 activities favored the formation of Cd-rich sphalerite (Curves 2 and 3 in Fig. 3). This result was consistent with the ore-forming conditions of MVT and SEDEX deposits. MVT deposits commonly form at temperatures <250 °C (typically between 90 and 150 °C) and salinities of 15–35% in oxidized (SO_4_^2−^-dominant) and acidic to near-neutral brines evolved from carbonate-dominated sedimentary basins^2^. In contrast, SEDEX deposits commonly form at temperatures of 120–250 °C and variable salinities (10–30 wt%) from acidic, reduced (H_2_S-predominant) connate brines evolved from reduced siliciclastic and shale basins^7^. Therefore, it is reasonable that fundamental differences in the chemistry of the mineralizing fluid for MVT and SEDEX deposits will result in distinct Cd distributions in sphalerite.

The highest K_T_ values were found to be nearly identical for all physico-chemical conditions, especially when the temperature reached 300 °C, indicating that high temperatures favor Cd substitution in sphalerite under hydrothermal conditions. However, studies on the solubility of sphalerite have shown that temperature may be one of most important factors controlling Zn deposition. For example, Tagirov and Seward^34^ have found that the solubility of sphalerite at 300 °C is four orders of magnitude higher than that at 150 °C in a solution with pH = 4 and m_(NaCl)_ = 2, thus indicating that even in high-temperature systems, substantial sphalerite accumulation will occur only when the temperature decreases, as shown in Fig. 3.

Therefore, the distribution of Cd in sphalerite from various types of Pb-Zn deposits is the result of the competing influences of various parameters, according to the thermodynamic theory on the liquid-solid partitioning of Cd and Zn. The inherent differences in the physico-chemical conditions under which hydrothermal fluids form various types of Pb-Zn deposits are key factors resulting in different Cd concentrations and Zn/Cd ratios in sphalerite.

### Mechanism underlying Cd isotopic variations in Pb-Zn deposits:

Because of their similar chemical and crystallographic properties, Cd closely follows Zn in geochemical cycling^11^, and thus, the isotope fractionation of these two elements may be similar^37^. Therefore, several causes analogous to those for Zn isotopes are proposed to explain the mass-dependent Cd isotopic variations in different systems, including (1) the equilibrium effect, (2) the kinetic effect, and (3) the Cd source.

Many studies have shown that evaporation and condensation processes can yield large Cd isotopic fractionation in meteorite samples with δ^114/110^Cd values ranging from −8‰ to +16‰^16^,^17^. Recently, large Cd isotopic fractionations have been reported for partly evaporated, industrially produced Cd metal, yielding a total δ^114/110^Cd fractionation of approximately 1‰^38^,^39^. However, these processes are associated with the gas transformation of solid Cd minerals, and homogeneous isotopic compositions of samples from high-temperature systems suggest that gas transformation may not occur during mineralization. Yang *et al.*^37^ have reported a systematic approach using quantum chemical calculations (density functional theory) of Cd isotope reduced partition function ratios to understand the fractionation properties of Cd species in hydrothermal fluids. Considering cadmium hydrate, chloride, hydroxide, nitrate and hydrosulfide complexes, Yang *et al.* have found that Cd isotopes show higher fractionation at low temperatures than at high temperatures, at which light isotopes are enriched in the solid phase. This result strongly suggests that low-temperature processes lead to significant isotopic fractionation of Cd in nature, in agreement with the Cd variation in the three systems considered here.

**Figure 4:**
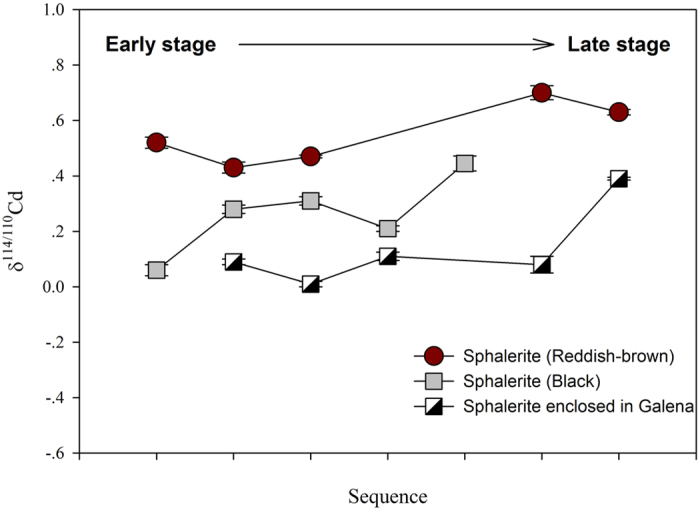
Cd isotopic variations in different early- to late-stage samples from the No. 78 orebody in the Fule MVT Pb-Zn deposit.

The Zn isotopes of Pb-Zn deposits, such as the Alexandrinka (VHMS-type), Irish Midlands (Irish-type), Red Dog (SEDEX-type) and Navan (Irish-type), have been studied^9^,^40^,^41^ and explained by Rayleigh distillation. However, only a few studies have considered Cd isotopes. A recent study^21^ has demonstrated that Cd readily replaces Ca during Cd sorption onto calcite. This process can be explained by Rayleigh fractionation using a fractionation factor ranging from 0.99943 to 0.99967. Such a process may occur during ore formation. A detailed study of the Fule MVT Pb-Zn deposit has revealed that Cd in early stages of sphalerite formation is enriched in light isotopes, whereas heavy isotopes become enriched in later stages (Fig. 4). In addition, at the same stage, the first precipitated sphalerite, enclosed in galena, is enriched with light Cd isotopes, in contrast to black sphalerite, which is crystallized a second time in galena. The reddish-brown sphalerite is the last to precipitate and presents the heaviest Cd isotope composition. Preferentially removing light Cd isotopes from the fluids may reveal a kinetic effect; however, the observed restricted fractionation favored a strong equilibrium effect as illustrated in Fig. 4.

Besides isotope fractionation during chemical reaction, the source-rock δ^144/110^Cd value may be another factor in Cd isotopic variability. Metals in the high-temperature system (porphyry, skarn and VMS deposits) are closely associated with magmatism, and although limited data exist, several igneous MORB and OIB basalt samples have revealed homogenous Cd isotopic compositions (δ^114/110^Cd value of 0.01 ± 0.06‰)^20^,^23^. Thus, the initial Cd isotopic compositions for high-temperature system fluids may be small or near zero. Sedimentary strata providing a source of metals in exhalative systems carry metal ions trapped within clay and phyllosilicate minerals and adsorbed onto mineral surfaces. These characteristics are consistent with shale-hosted SEDEX deposits having a large Cd isotopic variation: δ^114/110^Cd values of −0.48–0.12‰^23^. Finally, the sources of metals in MVT deposits may be complicated, but carbonate, which hosted MVT deposits, may provide an important contribution. Experimental studies have shown that Cd isotopic compositions of marine carbonates record a seawater Cd signature^21^. The global deep-ocean is typically enriched in heavy Cd isotopes with a mean δ^114/110^Cd value of 0.3‰^18^,^19^,^22^,^42^. Therefore, it is reasonable to assume that carbonate sources have heavy Cd isotopic compositions.

**Figure 5:**
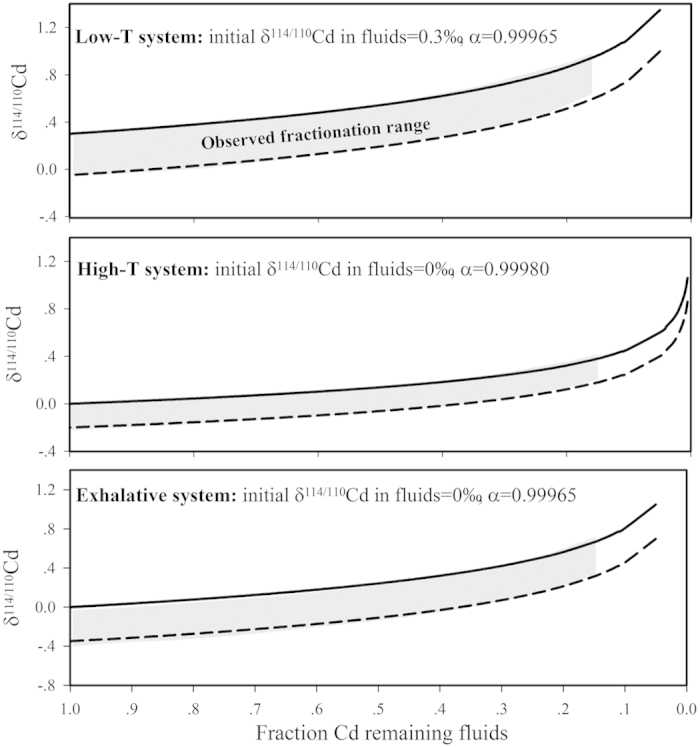
Evolution of δ^144/110^Cd during the deposition of aqueous Cd in different hydrothermal fluids. Dashed lines represent the evolution of the deposited minerals, and solid lines represent the evolution of residual aqueous Cd. The grey fields represent the observed fractionation range of the sphalerite samples.

Combining all the above factors, the Cd variation of the three systems can be explained using a Rayleigh distillation model, as illustrated in Fig. 5. The fractionation factor estimated herein is comparable to those determined previously^21^,^23^, and the high-temperature system probably provides a slightly lower fractionation factor, consistently with quantum chemical calculations (density functional theory), resulting in a restricted Cd isotope variation range^37^. The low-temperature and exhalative systems provide the same fractionation factor with a large dispersion of the Cd isotopic composition, whereas the δ^114/110^Cd values of the low-temperature systems are heavier, reflecting the difference in the original fluids resulting from their different Cd sources.

## Conclusions

**Figure 6:**
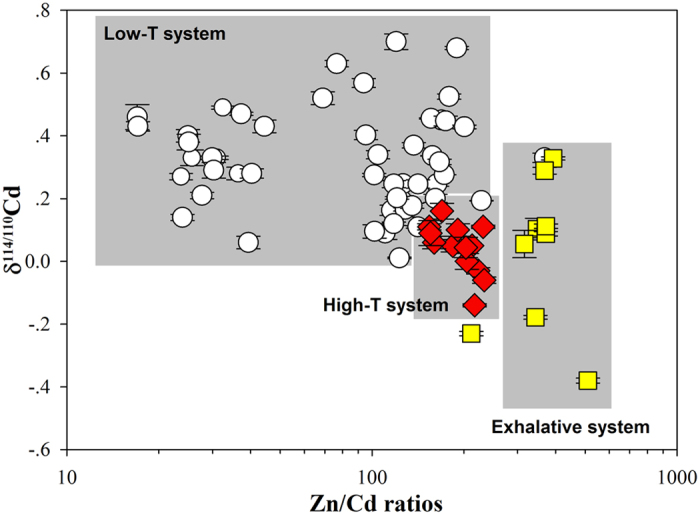
Distribution of Zn/Cd ratios versus Cd isotopic compositions in different mineralization systems.

Overall, this study provides some clarity regarding the mechanism by which Cd concentrations vary among Pb-Zn deposit types and variation in Zn/Cd ratios. Furthermore, the isotopic fraction outcomes of different geological and geochemical conditions are presented, and a schematic diagram is provided to develop the use of Cd isotopes as a proxy for the classification of Pb-Zn deposits (Fig. 6). Most importantly, theoretical considerations and field measurements are combined provide the first direct evidence that Cd distribution and isotopic fractionation in sphalerite, which are strongly dependent on the Cd source and the geochemical conditions of the ore-forming fluid (temperature, ΣS_red_ activities, pH and salinity), can be used as potential geochemical proxies to classify the corresponding Pb-Zn deposits.

## Methods

A total of 70 sphalerite samples were collected from these deposits and separated, and their Zn and Cd concentrations, and Cd isotopes were analyzed. The Zn and Cd concentrations were determined by inductively coupled plasma optical emission spectrometry (ICP-OES, Varian Vista MPX). The Cd isotope measurements were performed at the State Key Laboratory of Ore Deposit Geochemistry at the Institute of Geochemistry, Chinese Academy of Sciences, using a Neptune Plus multi collector ICP-MS instrument. An anion-exchange resin column was used to separate the Cd from the matrix. The method resulted in a mean Cd recovery of 99.8%. Elements that could potentially interfere with the determination of Cd isotopes, such as Sn, In, Zn, and Pb, were found at negligible concentrations relative to Cd. The standard–sample bracketing (SSB) method was used to calculate the delta values^43^. The delta values for Cd isotopes are expressed as: 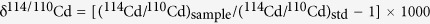, where “std” is the internal reference standard (Spex solution)^12^. Further details about the analytical methods and data are provided in the Supplementary materials.

## Additional Information

**How to cite this article**: Wen, H. *et al.* Zn/Cd ratios and cadmium isotope evidence for the classification of lead-zinc deposits. *Sci. Rep.* **6**, 25273; doi: 10.1038/srep25273 (2016).

## Supplementary Material

Supplementary Information
