# Supplementary material for: Zn/Cd ratios and cadmium isotope evidence for the classification of lead-zinc deposits
Source: Sci Rep. 2016 Apr 28;6:25273. doi: 10.1038/srep25273 (PMC4848528; doi:10.1038/srep25273)
Supplement: Supplementary Information [file srep25273-s1.pdf]

Zn/Cd ratios and cadmium isotope evidence for the classification of lead-zinc deposits

Hanjie Wen[1], Chuanwei Zhu[1], Yuxu Zhang[1], Christophe Cloquet[2], Haifeng Fan[1], Shaohong Fu[1]

[1] State Key Laboratory of Ore Deposit Geochemistry (SKLODG), Institute of Geochemistry, Chinese Academy of Sciences, Guiyang, 550002, China

[2] Centre de Recherches Petrographique et Geochimiques, CNRS/UMR 7358, 15, Rue Notre-Dame-Pauvres, B. P. 20, 54501, Vandoeuvre-les-Nancy Cedex, France

Section 1. Ore deposit geology

Nine Pb-Zn deposits were chosen to investigate Cd isotopic compositions and relevant elemental ratios. These were divided into three categories: high-temperature systems, low-temperature systems and exhalative systems. The high-temperature system includes a porphyry deposit (Dabaoshan), magmatic hydrothermal deposit (Shagou), skarn deposit (Bayinnuoer) and VMS deposit (Gacun), all of which are closely associated with magmatism and formed at temperatures of 200–250 ℃ (Han et al., 2014; Li et al., 2012; Sun, 1992; Tornos et al., 2015). Low-temperature systems refer to MVT deposits, including the Fule, Tianbaoshan, Jinding and Dadongla Pb-Zn deposits, typically formed at temperatures < 200 ℃ (Zaw et al., 2007). Finally, exhalative systems are represented by the Langshan SEDEX deposit. Ore deposit geology for these deposits are provided in Table 1 as following.

Table 1-1 Characteristics of selected Pb-Zn deposits

Deposit	Fule	Tianbaoshan	Jinding	Dadongla
Deposit type	MVT	MVT	MVT	MVT
Province/tectonic unit	E. Guizhou, Yangtze Craton	W.Sichuan, Yangtze Craton	W.Yunnan, Sanjiang Fold Systerm	E. Guizhou, Yangtze Craton
Tonnage(Mt)	0.38 (Mt): combined (Zn+Pb+Cd+Ge+Ga)	2.6 (Mt): combined (Zn+Pb+Ag)	200 Mt (ore): combined (Zn+Pb+Ag)	3.2×10^{-3}(Mt): combined(Zn+Hg)
Grade	4.51% Zn, 0.56% Pb, 0.13% Cd, 0.01% Ge, 0.007% Ga	10.4% Zn, 1.4% Pb, 93.6 g/t Ag	6.08% Zn, 1.29% Pb, 1-20 g/t Ag	No data
Orebody morphology	Massive stratifom, lens-shaped	Massive, disseminated	Tabular, vein, massive stratiform	disseminated, massive
Host lithology	Early Permian，Maikou Fm.(dolomite, limestone)	Dengying Fm.(dolomite, limestone)	Early Cretaceous, Jinxing Fm.(sandstone, limestone) Tertiary sediments	Early Cambrian, Aoxi Fm.(dolomite)
Mineralisation age (Ma)	108-198	Earlier than 166-156	72±4.4	101±5.5
ore-forming temperature	160℃-220℃(Zn-Pb ore related)	120℃-160℃(Zn-Pb ore related)	100℃-210℃	130℃-170℃
Salinity	4.5-11 wt%	6.2-7.4 wt%	5-10 wt%	15-25 wt%

Table 1-2 Characteristics of selected Pb-Zn deposits

Deposit	Dabaoshan	Shagou	Baiyinnuo'er	Gacun	langshan
Deposit type	Porphyry type	magmatic hydrothermal type	Skarn type	VMS type	Sedex
Province/tectonic unit	N.Guangzhou, Cathaysia Block	NW.Henan, North China craton	E.Inner Mongolia,North China craton	W.Sichuan, Sanjiang Fold Systerm	northern margin of North China craton
Tonnage(Mt)	2.1 (Mt): combined (Cu+Zn+Pb+W)	1.54 (Mt): combined (Ag-Pb-Zn)	2.44 (Mt): combined (Zn+Pb+Ag)	4 (Mt): combined (Cu+Zn+Pb+Ag+Au)	6 (Mt): combined (Zn+Pb)
Grade	0.86% Cu, 12% Zn, 1.77% Pb, 0.12% WO_3	13.24% Pb, 4.31% Zn, 767 g/t Ag	5.73-8.67% Zn, 1.72-2.09%, Ag (1027 t)	0.44% Cu, 5.4% Zn, 3.7% Pb, 160 g/t Ag, 0.3 g/t Au	3.95% Zn, 1.35% Pb
Orebody morphology	Massive stratifom, lens-shaped	Disseminated, massive	Disseminated	Massive stratifom	Massive stratifom
Host lithology	Middle Devonian-Carboniferous, Donggangling Fm. (limestone)	Early Archaeozoic-Proterozoic , Taihua Gr and Xionger Gr. (volcanics)	Early Permian Huangliang Fm.(Limestone, marble)	Late Triassic, Gacun Fm.(volcanics)	Mesoproterozoic Zhaertaishan Group，(dolomite)
Mineralisation age (Ma)	166±3.0	140.0±1.0	244.5±0.9	204±14	1400-1600
ore-forming temperature	280℃-320℃ (Zn-Pb ore related)	194℃-240℃ (Zn-Pb ore related)	197℃-373℃	122℃-250℃ (Zn-Pb ore related)	160℃-320℃ (Zn-Pb ore related)
Salinity	2-17 wt%	1.9-10.9 wt%	1.9-10.9 wt%	0.5-10.7 wt%	8-15 wt%

Section 2. Chemistry and mass spectrometry

A total of 70 sphalerite samples from these deposits were collected, separated and analyzed for Zn and Cd concentrations and Cd isotopes. The Zn and Cd concentrations were determined by Inductively Coupled Plasma Optical Emission Spectrometry (ICP-OES, Varian Vista MPX) at the State Key Laboratory of Ore Deposit Geochemistry, at the Institute of Geochemistry, Chinese Academy of Sciences

After the Cd concentrations in the powder samples were determined, an aliquot that would have contained more than 100 ng Cd was taken from each sample and oxidized using a 3 ml of concentrated HNO_3 at 100 ℃ for at least 16 h, until the

sample was completely dissolved. A procedure using an anion exchange resin column, which has been described by (Cloquet et al., 2005; Wen et al., 2015; Zhu et al., 2013), was used to separate the Cd from the matrix. The procedure that was used to separate the Cd from the matrix is shown in **Table 2**. The method gave a mean Cd recovery of 99.8%. Elements that could potentially interfere with the determination of the Cd isotopes, such as Sn, In, Zn, and Pb, were found at concentrations that were negligible relative to the Cd concentrations.

Table 2 Summary of the Cd separation Chemistry

Procedure	Reagent	volume (ml)
Resin	AG-MP-1M (100-200 mesh)	3
Clean	0.0012 mol/L HCl	25
Condition	2 mol/L HCl	20
Load sample	2 mol/L HCl	2
Wash	2 mol/L HCl	10
Wash	0.3 mol/L HCl	30
Wash	0.06 mol/L HCl	20
Wash	0.012 mol/L HCl	10
Collect Cd	0.0012 mol/L HCl	20

The Cd isotope measurements were performed at the State Key Laboratory of Ore Deposit Geochemistry, at the Institute of Geochemistry, Chinese Academy of Sciences, using a Neptune Plus multi collector ICP-MS instrument. Aliquots of the digested samples were introduced into the instrument using a cyclonic chamber system with a perfluoroalkoxy alkane pneumatic nebulizer in free aspiration mode. The samples and bracketing reference solutions were run in two blocks of 15 measurement cycles for each m/z ratio. The system typically generated a total Cd signal of about 57 V/ppm at an uptake rate of about 100 μL/min, which corresponded to ca. 75 ng Cd analyzed. The nebulizer and spray chamber were rinsed after each run with 0.6 M HNO_3 until the signal intensity reached the original background level

(generally after 3 min).

The standard–sample bracketing method was used to calculate delta values. The concentrations in the samples and reference Cd samples (the bracketing Cd reference was obtained from Spex) were matched to within 10%. The analyses were conducted in static mode. The blank solution signal was subtracted from each of the measured masses. Instrumental drift was corrected by averaging the ratios measured in the bracketing reference solutions. Only sections with linear or smooth drifts for the reference solution were considered and used to calculate the delta values for the samples. A Cd solution (Spex) was used as an internal reference standard. A fractionated Cd metal sample (a Münster Cd solution from Nancy University) was used as a second reference material. Repeated measurements of the Münster Cd solution gave a $\delta^{114/110}Cd$ value of 4.47‰ ± 0.08‰ (mean ± 2σ), which was identical to the previously recommended Cd isotopic value for the solution, 4.48‰ (Cloquet et al., 2005). δ notation, as defined by the relationship shown below, was used to present the results.

$$\delta^{x/110}Cd\ (‰) = [({}^{x}Cd/{}^{110}Cd)_{sample}/({}^{x}Cd/{}^{110}Cd)_{std} - 1] \times 1000‰,$$

where ${}^{x}Cd$ means the ${}^{111}Cd$, ${}^{112}Cd$, ${}^{113}Cd$, or ${}^{114}Cd$ isotope.

The $\delta^{112/110}Cd$ vs $\delta^{114/110}Cd$ diagram (Fig. 1) shows that the Cd isotopic compositions of all sphalerite samples fall within the error for the equilibrium theoretical mass fractionation lines, indicating that isobaric interference has been efficiently removed by the chemical purification (matrix interference) and by the isobaric interference correction (e.g., Sn interference) during the measurements.

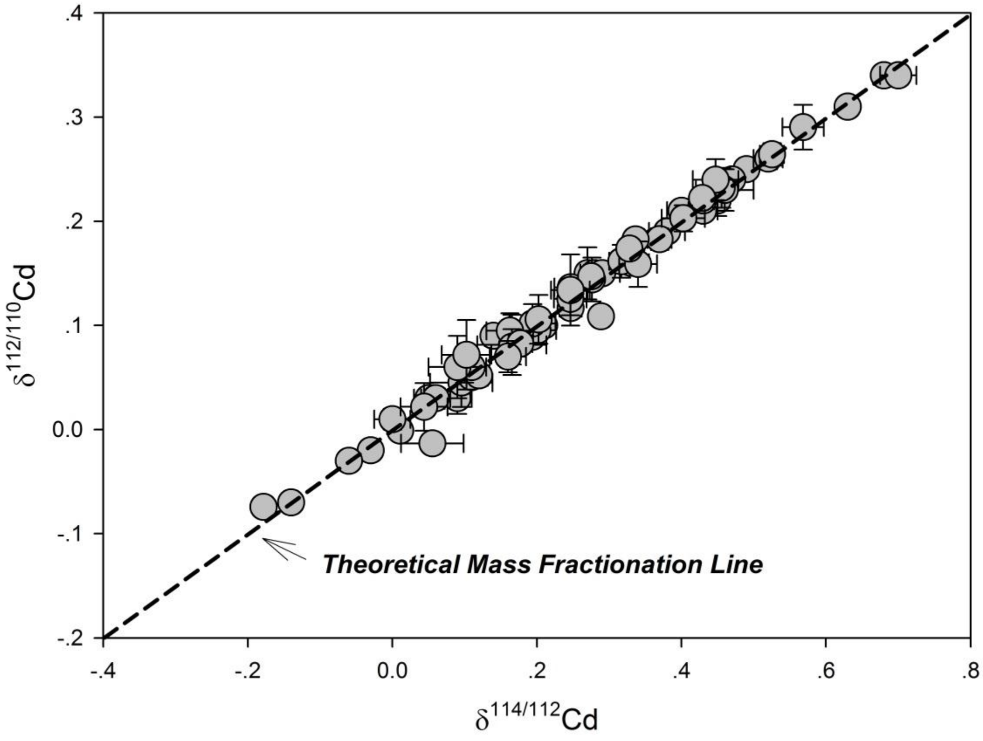

Figure 1 A $\delta^{114/110}$Cdsp vs $\delta^{112/110}$Cd diagram for the samples from the studied deposits, and all samples are within the error of the theoretical mass fractionation line (TMFL).

Section 3. Dataset of the studied deposits

Table 3 The analytical data with elemental analyses, elemental enrichment ratios (Zn/Cd) and isotopic values from both the studied deposits and reference data

Sample No.	Sample type	Cd (ppm)	Zn (%)	Fe (ppm)	Zn/Cd	$\delta^{114/110}$Cd (2σ, ‰)	$\delta^{112/110}$Cd (2σ, ‰)
**Low-temperature system**							
Dadongla MVT Pb-Zn deposit							
DDL13-2	Sp, Reddish-brown	26215	62.0		24	0.27±0.02	0.15±0.05
DDL13-6	Sp, Reddish-brown	16536	60.2		36	0.28±0.04	0.15±0.01
DDL14-3	Sp, Reddish-brown	22857	58.7		26	0.33±0.05	0.16±0.02
DDL14-3	Sp, Reddish-brown	19090	59.2		31	0.33±0.01	0.16±0.02
DDL14-5	Sp, Reddish-brown	20073	65.1		32	0.49±0.01	0.25±0.01
Jinding MVT Pb-Zn deposit							
JD-13	Sp, Black	3208	54.1		169	0.45±0.02	0.22±0.03
JD-13	Sp, Buff	3184	60.3		189	0.68±0.01	0.34±0.02
JD2-10	Sp, Buff	5496	60.6		110	0.09±0.04	0.03±0.03

JD3-16	Sp, Black	22826	54.6		24	0.14±0.02	0.09±0.02
JD3-9	Sp, Black	4805	66.3		138	0.33±0.03	0.16±0.02
Fule MVT Pb-Zn deposit							
SBFL-16	Sp, Mixed	22151	55.2	369	24.9	0.40±0.04	0.21±0.01
SBFL-16	Sp, Mixed	22064	55.3	419	25.0	0.38±0.05	0.19±0.01
SBFL-22	Sp, Black	14714	57.9	970	39.3	0.06±0.04	0.03±0.01
SBFL-22	Sp, Reddish-brown	9083	62.6	448	69.0	0.52±0.04	0.26±0.02
SBFL-23	Sp, Black	15046	60.7	706	40.3	0.28±0.03	0.15±0.01
SBFL-23	Sp, Reddish-brown	13735	60.9	685	44.3	0.43±0.04	0.21±0.01
SBFL-24	Sp, Black	18479	55.3	547	29.9	0.33±0.01	0.17±0.01
SBFL-24	Sp, Reddish-brown	16783	62.5	365	37.2	0.47±0.01	0.24±0.01
SBFL-24	Sp, Black	19532	59.2	450	30.3	0.29±0.05	0.15±0.01
SBFL-25	Sp, Black	18645	51.7	446	27.7	0.21±0.02	0.10±0.02
SBFL-26	Sp, Black	34981	59.5	395	17.0	0.46±0.08	0.23±0.04
SBFL-26	Sp, Black	34757	59.4	384	17.1	0.43±0.03	0.22±0.01
FL-01	Sp, Reddish-brown	5238	62.8	424	119.9	0.70±0.05	0.34±0.01
FL-04	Sp, Reddish-brown	7930	60.7	492	76.6	0.63±0.02	0.31±0.01
Fule MVT Pb-Zn deposit							
HL-6-2	Sp, Black	4524	42.5	13474	93.8	0.57±0.06	0.29±0.04
HL-6-3	Sp, Black	2798	45.6	13163	163.1	0.25±0.03	0.12±0.01
HL-6-4	Sp, Black	2745	42.8	12463	156.1	0.46±0.01	0.23±0.04
HL-8-3	Sp, Black	2534	44.1	12297	174.2	0.45±0.06	0.24±0.04
HL-8-4	Sp, Buff	2415	48.5	3632	200.7	0.43±0.02	0.22±0.01
HL-8-4	Sp, Black	2608	46.6	11428	178.8	0.53±0.03	0.26±0.02
HL-8-5	Sp, Black	1998	45.6	11004	228.1	0.19±0.02	0.09±0.01
HL-8-10	Sp, Black	4266	43.5	13495	101.9	0.10±0.09	0.05±0.05
HL-6-3	Sp, Black	2798	45.1	13129	161.1	0.20±0.05	0.10±0.02
HL-8-16	Sp, Buff	3100	39.3	8430	126.6	0.17±0.10	0.08±0.06
HL-8-13	Sp, Black	3348	41.9	13304	125.0	0.19±0.06	0.10±0.04
HL-7-3	Sp, Reddish-brown	4270	49.5	8103	115.9	0.16±0.06	0.09±0.03
HL-7-2	Sp, Black	3488	44.8	9375	128.5	0.17±0.02	0.08±0.03
HL-7-25	Sp, Black	3273	46.3	12168	141.3	0.11±0.02	0.05±0.01
HL-7-2	Sp, Reddish-brown	4113	48.5	8546	117.8	0.12±0.02	0.05±0.02
HL-7-26	Sp, Black	3273	40.2	8567	122.9	0.01±0.01	0.00±0.01
HL-7-6	Sp, Black	3445	46.6	12437	135.4	0.18±0.03	0.08±0.01
HL-7-23	Sp, Black	3354	45.9	12512	136.9	0.37±0.03	0.18±0.02
HL-7-22	Sp, Black	2474	42.7	10397	172.5	0.28±0.00	0.14±0.04
HL-7-8	Sp, Black	3591	45.3	9715	126.3	0.25±0.05	0.14±0.00
HL-7-19	Sp, Reddish-brown	3091	48.7	11015	157.6	0.34±0.02	0.18±0.00
HL-7-19	Sp, Black	2721	45.2	12686	166.2	0.32±0.03	0.16±0.03
HL-7-1	Sp, Black	4296	44.9	8540	104.5	0.34±0.05	0.16±0.04
HL-7-3	Sp, Black	4402	44.7	8964	101.6	0.28±0.02	0.15±0.01
HL-7-1	Sp, Reddish-brown	4111	48.4	7958	117.7	0.25±0.05	0.13±0.03
HL-7-10	Sp, Black	3320	46.9	10554	141.4	0.25±0.05	0.13±0.07

HL-8-16	Sp, Black	3058	36.9	7874	120.7	0.20±0.02	0.11±0.05
HL-8-16	Sp, Black	4887	46.7	12746	95.5	0.40±0.06	0.20±0.03
**High-temperature system**							
Bayinnuoer skarn deposit							
BYNE-1	Sp, Black	2521	54.7		217	-0.14±0.01	-0.07±0.01
BYNE-2	Sp, Buff	2627	58.5		223	-0.03±0.02	-0.02±0.01
BYNE-3	Sp, Buff	2483	57.9		233	-0.06±0.02	-0.03±0.01
BYNEr-3	Sp, Buff	2410	55.7		231	0.11±0.01	0.06±0.01
Gacun VMS deposit							
Gacun-1	Sp, Reddish-brown	2926	62.2		213	0.05±0.00	0.03±0.02
Gacun-2	Sp, Reddish-brown	3041	51.5		169	0.16±0.05	0.07±0.03
Gacun-3	Sp, Reddish-brown	2828	57.8		204	0.00±0.05	0.01±0.02
Gacun-4	Sp, Black	3476	63.9		184	0.05±0.04	0.03±0.02
Shagou magmatic hydrothermal deposit							
SG-92	Sp, Black	2688	51.3		191	0.10±0.04	0.06±0.03
SG-96	Sp, Buff	4126	63.6		154	0.11±0.02	0.06±0.01
SG-96	Sp, Black	3819	61.0		160	0.06±0.04	0.03±0.02
SG-96	Sp, Buff	3856	59.9		155	0.09±0.08	0.06±0.06
Dabaoshan porphyry deposit							
DBS-13	Sp, Buff	1316	26.7		203	0.04±0.06	0.02±0.05
**Exhalative system**							
Langshan SEDEX deposit							
LS-1	Sp, Buff	996	34.2		343	-0.18±0.01	-0.07±0.01
LS-2	Sp, Buff	904	31.4		347	0.10±0.07	0.07±0.07
TYK-1	Sp, Buff	595	23.4		393	0.33±0.01	0.17±0.01
TYK-8	Sp, Buff	34	1.1		316	0.06±0.09	-0.01±0.01
**Seafloor hydrothermal sulfides (Schmitt et al., 2009)**							
JSPX-1	Sp, Buff	31	1.1		368	0.29±0.02	0.11±0.01
Alv-4053-M1 #A1 (bottom chimney)	Sphalerite+pyrite	1174	25		211	-0.23±0.01	
Alv-4053-M1 #A5 (top chimney)	Sphalerite+pyrite	259	13		510	-0.38±0.02	
Alv-4053-M2 -a (extinct)	Euhedral sphalerite					0.46±0.01	
Alv-4057-M1 #A1 (bottom chimney)	Sphalerite+pyrite	611	23		371	0.09±0.01	
Alv-4057-M1 #A4 (top chimney)	Sphalerite+pyrite	714	27		371	0.11±0.01	
Alv-4059-M3	Pyrite+sphalerite					0.11±0.01	

NL-10-04	Sphalerite+barite	0.16±0.04

Reference

Cloquet, C., Rouxel, O., Carignan, J., and Libourel, G., 2005, Natural Cadmium isotopic variations in eight geological reference materials (NIST 2711, BCR 176, GSS 1, GXR 1, GXR 2, GSD 12, Nod P1, Nod A1) and anthropogenic samples, measured by MC-ICP MS: Geost. Geoan. Res. , v. 29, p. 95-106.

Han, J.-S., Yao, J.-M., Chen, H.-Y., Deng, X.-H., and Ding, J.-Y., 2014, Fluid inclusion and stable isotope study of the Shagou Ag–Pb–Zn deposit, Luoning, Henan province, China: Implications for the genesis of an orogenic lode Ag–Pb–Zn system: Ore Geology Reviews, v. 62, no. 0, p. 199-210.

Li, C.-Y., Zhang, H., Wang, F.-Y., Liu, J.-Q., Sun, Y.-L., Hao, X.-L., Li, Y.-L., and Sun, W., 2012, The formation of the Dabaoshan porphyry molybdenum deposit induced by slab rollback: Lithos, v. 150, no. 0, p. 101-110.

Schmitt, A. D., Galer, S. J. G., and Abouchami, W., 2009, Mass-dependent cadmium isotopic variations in nature with emphasis on the marine environment: Earth and Planetary Science Letters, v. 277, no. 1-2, p. 262-272.

Sun, H., 1992, A general review of volcanogenic massive sulphide deposits in China: Ore Geology Reviews, v. 7, no. 1, p. 43-71.

Tornos, F., Peter, J. M., Allen, R., and Conde, C., 2015, Controls on the siting and style of volcanogenic massive sulphide deposits: Ore Geology Reviews, v. 68, no. 0, p. 142-163.

Wen, H., Zhang, Y., Cloquet, C., Zhu, C., Fan, H., and Luo, C., 2015, Tracing sources of pollution in soils from the Jinding Pb–Zn mining district in China using cadmium and lead isotopes: Applied Geochemistry, v. 52, p. 147-154.

Zaw, K., Peters, S. G., Cromie, P., Burrett, C., and Hou, Z., 2007, Nature, diversity of deposit types and metallogenic relations of South China: Ore Geology Reviews, v. 31, no. 1–4, p. 3-47.

Zhu, C. W., Wen, H. J., Zhang, Y. X., Fan, H. F., Fu, S. H., Xu, J., and Qin, T. R., 2013, Characteristics of Cd isotopic compositions and their genetic significance in the lead-zinc deposits of SW China: Science China-Earth Sciences, v. 56, no. 12, p. 2056-2065.

Zhu, C. W., Wen, H. J., Zhang, Y. X., Fan, and H. F., 2016, Cadmiumand sulfur isotopic compositions of the Tianbaoshan Zn–Pb–Cd deposit, Sichuan Province, China: Ore Geology Reviews, v. 76, p. 152–162
